# Gene Annotation and Transcriptome Delineation on a De Novo Genome Assembly for the Reference *Leishmania major* Friedlin Strain

**DOI:** 10.3390/genes12091359

**Published:** 2021-08-29

**Authors:** Esther Camacho, Sandra González-de la Fuente, Jose C. Solana, Alberto Rastrojo, Fernando Carrasco-Ramiro, Jose M. Requena, Begoña Aguado

**Affiliations:** Centro de Biología Molecular “Severo Ochoa” (CBMSO, CSIC-UAM), Campus de Excelencia Internacional (CEI) UAM+CSIC, Universidad Autónoma de Madrid, 28049 Madrid, Spain; ecamacho@cbm.csic.es (E.C.); sandra.g@cbm.csic.es (S.G.-d.l.F.); jcsolana@cbm.csic.es (J.C.S.); arastrojo@cbm.csic.es (A.R.); fcarrasco@cbm.csic.es (F.C.-R.); baguado@cbm.csic.es (B.A.)

**Keywords:** genome, transcriptome, gene models, *Leishmania*, Illumina sequencing, PacBio sequencing, expression levels, untranslated regions (UTR), SL-additions site (SAS), polyadenylation site (PAS)

## Abstract

*Leishmania major* is the main causative agent of cutaneous leishmaniasis in humans. The Friedlin strain of this species (LmjF) was chosen when a multi-laboratory consortium undertook the objective of deciphering the first genome sequence for a parasite of the genus *Leishmania*. The objective was successfully attained in 2005, and this represented a milestone for *Leishmania* molecular biology studies around the world. Although the LmjF genome sequence was done following a shotgun strategy and using classical Sanger sequencing, the results were excellent, and this genome assembly served as the reference for subsequent genome assemblies in other *Leishmania* species. Here, we present a new assembly for the genome of this strain (named LMJFC for clarity), generated by the combination of two high throughput sequencing platforms, Illumina short-read sequencing and PacBio Single Molecular Real-Time (SMRT) sequencing, which provides long-read sequences. Apart from resolving uncertain nucleotide positions, several genomic regions were reorganized and a more precise composition of tandemly repeated gene loci was attained. Additionally, the genome annotation was improved by adding 542 genes and more accurate coding-sequences defined for around two hundred genes, based on the transcriptome delimitation also carried out in this work. As a result, we are providing gene models (including untranslated regions and introns) for 11,238 genes. Genomic information ultimately determines the biology of every organism; therefore, our understanding of molecular mechanisms will depend on the availability of precise genome sequences and accurate gene annotations. In this regard, this work is providing an improved genome sequence and updated transcriptome annotations for the reference *L. major* Friedlin strain.

## 1. Introduction

Leishmaniasis is a group of neglected tropical diseases caused by parasitic protists of the genus *Leishmania*. This parasite has a digenetic life cycle, alternating between the alimentary tract of the sandfly vector, as an extracellular promastigote, and the phagolysosomal vacuole of macrophages, in which the parasite adopts the amastigote form. Transmissions to humans occur in nearly 100 countries, and around one million new cases of leishmaniasis are reported per year [1]. Unfortunately, there is no effective vaccine for the prevention of human leishmaniasis [2], and the current treatments are based on chemotherapy, which relies on four drugs having problems of toxicity, cost, growing drug resistance, and/or treatment failure [3]. 

Given the global relevance of leishmaniasis, in 1994, the WHO Leishmania Genome Initiative was launched, bringing together a large number of laboratories determined to sequence the full genome of a pathogenic *Leishmania* species [4]. *Leishmania major* was the selected one and the genome sequence was determined on a chromosome-by-chromosome basis. Firstly, a genome physical map was constructed from 9216 genomic cosmids by DNA hybridizations using probes derived from the ends of contigs and chromosome specific probes [5]. Meanwhile, contigs were fragmented and sequenced by the classical Sanger’s sequencing technique. After sequence assembling, the accuracy of sequence assemblies was assessed by comparison to optical maps for the 36 chromosomes of *L. major* genome [6]. Finally, in 2005, the complete genome sequence and gene annotations were reported [7]. This work represented a milestone that provided important insights about the gene content and genome architecture of this parasite and paved the way for genome-wide studies [8]. Soon after, the genome sequences for two other *Leishmania* species, *Leishmania infantum* and *Leishmania braziliensis,* were produced by whole-genome shotgun cloning and classical Sanger’s sequencing [9], even though these assemblies did not achieve the completeness of that attained for the *L. major* genomic assembly. 

The development of next generation sequencing (NGS) technologies has transformed the field of genomics, and genome sequencing became an affordable and indispensable technique for molecular biology studies. Hence, a continuously growing number of genomes are being sequenced and, particularly, within the genus *Leishmania*, most of the named species have their genome sequenced [10,11,12,13,14,15,16,17,18]. Remarkably, due to its high quality, the 2005-genome sequence of *L. major* Friedlin has remained as the reference genome and has been used for the template-guided assembly of those new sequenced genomes. However, despite its relevance, the *L. major* (Friedlin) genome assembly cannot be considered as a final product ‘set on stone’. In fact, previous studies have documented some deficiencies in this assembly [19,20]. Moreover, two features of the *Leishmania* genomes represent hurdles for a correct assembly based on sequencing short DNA fragments. On the one hand, the existence of a large number of repetitive DNA sequences, which are scattered along the different *Leishmania* chromosomes [21,22,23], is the cause of conflict for assemblers. On the other hand, many loci in *Leishmania* genomes are comprised of multiple identical gene copies that are head-to-tail tandemly arranged [24]; in this case, assembly collapses lead to underestimation on the real number of gene copies. The third-generation/long-read sequencing methods have solved most of these issues, contributing to produce genome assemblies of unprecedented quality [25]. However, a drawback of these new sequencing methods is the high sequence error rate, around 15% [25]. Thus, a combination of high-accurate short-reads and less-accurate long-reads has allowed the production of new and improved genomes assemblies for several *Leishmania* species [26,27,28,29,30,31].

The generation of high-quality genome assemblies is a basic step in the process of studying molecular mechanisms of gene expression, but additional information other than nucleotide sequences needs to be generated. There are dedicated bioinformatics tools, like Companion [32] that automatically and efficiently performs predictions of open reading frames (ORFs). However, protein-coding genes contain sequences other than ORFs, i.e., they also contain 5′- and 3′-untranslated regions (5′- and 3′-UTRs). Although some bioinformatics algorithms have been developed to delineate UTRs in *Leishmania* genes [33], the absence of conserved sequence motifs in the *Leishmania* gene boundaries precludes an accurate prediction of gene models, which only can be generated after obtaining the complete sequence of their transcripts. To date, experimental transcriptomes have been reported for *L. major* [34], *L. mexicana* [35], and *L. donovani* [30]. Genome wide gene expression studies require precise gene models, being especially relevant in *Leishmania*, where a significant number of genes share identical ORFs but differ substantially in their UTRs [36,37,38,39,40,41,42]. 

In this work, the *L. major* (Friedlin) genome was re-sequenced using the Pacific Biosciences (PacBio) technology, which provides long reads able to span long repeats, and the Illumina technology to generate paired-end short-reads useful to join fragmented chromosomes, extend chromosomes ends and correct homopolymer indel errors. As a result, here is reported the complete and improved sequence of the 36 chromosomes comprising the *L. major* genome. Additionally, based on this improved genome, a re-annotation of the *L. major* transcriptome is provided. This is a valuable information aimed to guide future studies on gene expression in this parasite.

## 2. Materials and Methods

### 2.1. Leishmania Parasites and DNA Isolation

Promastigotes of *L. major* (MHOM/IL/80/Friedlin) clone V1 were provided in 2004 by Dr. David Sacks (Laboratory of Parasitic Diseases, NIH, Bethesda, MD, USA). The parasites were grown at 26 °C in M199 medium supplemented with foetal bovine serum (10%), HEPES (40 mM; pH 7.4), adenine (0.1 mM), hemin (10 µg/mL), biotin (1 µg/mL) biopterin (2 ng/mL), penicillin G (100 U/mL), and streptomycin sulphate (0.1 mg/mL). 

The DNA for Illumina sequencing was prepared from 2 × 10^8^ promastigotes using the “High Pure PCR Template Preparation Kit” (Roche Diagnostics, Mannheim, Germany), following manufacturer’s instructions. DNA for PacBio sequencing was prepared from a similar number of promastigotes but following a classical phenol extraction method [43].

### 2.2. Illumina Sequencing

Library construction and paired-end sequencing were performed at the Centro Nacional de Análisis Genómico (CNAG-CRG, Spain; http://www.cnag.crg.eu/ accessed on 5 November 2015) using Illumina HiSeq 2000 technology. A total of 52,845,525 paired-end, 2 × 126 nucleotides (nt) sequence reads were generated. A median insert size of 305-bp was estimated. The reads were analyzed using PrinseqQuality (http://prinseq.sourceforge.net/ accessed on 3 March 2016) and poor-quality reads (cut-off value, 20) were removed; additionally, only those reads having a length ≥60-nt were considered. The filtered reads were assembled using the CLC Genomics Workbench version 5.0 (CLC Bio).

### 2.3. PacBio Sequencing and De Novo Assembly

The single-molecule real-time (SMRT) sequencing technology developed by PacBio [44] was used for generating long sequencing reads. A total of 285,082 pre-filtered reads were obtained on a PacBio RS II sequencing instrument. The Norwegian Sequencing Centre (www.sequencing.uio.no 5 October 2017) provided the sequencing service.

A hierarchical genome-assembly process (HGAP) [45], using the HGAP3 (Pacific Biosciences, SMRT Analysis Software v2.3.0) and HGAP4 (Pacific Biosciences, SMRT Link 4.0.0) protocols⁠, was carried out. Three strategies in the *de novo* genome assemblies were followed: (i) and (ii) HGAP3 and expected genome sizes of 34 and 35 Megabases (Mb), respectively, and (iii) HGAP4 and an expected genome size of 35 Mb. Equivalent assemblies were obtained in all three strategies. PacBio contigs having low coverage (< 40×) or short length (<15-Kb) were considered spurious and discarded.

### 2.4. Assembly Refinements

The assembled contigs were compared by BLAST [46] against the reference *L. major* Friedlin genome sequence (Tritryp, v.46). Thirty-one of the PacBio contigs represented complete chromosomes. The other five chromosomes were assembled in two PacBio contigs each. Minimus2 software [47], which is based on NUCmer algorithm, was used to compute overlaps between contigs in order to join these contigs into a sole chromosome. 

Additionally, Illumina contigs were aligned against these PacBio assembled chromosomes using LAST aligner (http://last.cbrc.jp/ accessed on 9 November 2017). These analyses served to extend the ends of chromosomes. For this purpose, three tools were used: MAFFT multiple-aligner [48], BLAST, and SSPACE-standard [49]. Finally, taking into account the distance information of paired-reads, GapCloser (https://sourceforge.net/projects/soapdenovo2/files/GapCloser/ accessed on 11 November 2017) and Gapfiller [50] were used to determine the appropriate size and sequence of the chromosomal extensions.

On the final assembly, a further sequencing revision was done by using ARAMIS [51], a recent tool developed to correct sequences derived from PacBio genome assemblies. For this purpose, Illumina reads were used and an indel fraction of 0.8 was selected.

In order to check assembling defects in genomic regions with unexpected frameshifts, we used SAMTools [52] to extract those reads mapping into a defined region. Afterwards, these reads were re-assembled using the Canu assembler, a tool specifically designed for noisy single-molecule sequences [53].

### 2.5. Coverage and Alignment

Coverage analyses on either the newly assembled chromosomes or the reference genome (LmjF) were performed using both Illumina and PacBio reads. Firstly, Illumina reads were aligned by Bowtie2 [54], and PacBio bax.h5 reads were aligned with BLASR [55]. Afterwards, coverage analysis was done from each alignment along the 36 chromosomes using the GenomeCoverageBed tool (http://bedtools.readthedocs.io/en/latest/content/tools/genomecov.html accessed on 12 November 2017) The graphical coverage plots files were generated using GNUPLOT (http://www.gnuplot.info/ accessed on 13 November 2017).

### 2.6. Somy Analysis

Somy estimation was performed using the 2-loop method, as described elsewhere [56]. Somy graphs were generated from the median coverage values for each chromosome using the bar plot function of the R package (https://cran.r-project.org accessed on 27 May 2019).

### 2.7. Synteny Analysis

Synteny was evaluated via progressive algorithm MAUVE [57] and genoPlotR [58] using as reference the *L. major* Friedlin genome in which the seven new loci identified by Alonso et al [20] were included. Gepard tool (https://academic.oup.com/bioinformatics/article/23/8/1026/198110 accessed on 3 May 2020) was used to create graphical plots for visualization of changes in synteny.

### 2.8. Haplotype Detection

The pre-processing of Illumina alignment file was carried out with Picard tools (http://broadinstitute.github.io/picard/ accessed on 17 June 2020) to reduce bias introduced by PCR amplification. GATK HaplotypeCaller (version 4.1) [59] was chosen to detect variants. The resulting VCF file was used to reconstruct individual haplotypes across the whole *L. major* Friedlin genome assembly by HapCUT2 [60], a maximum-likelihood-based tool designed for assembling haplotypes from DNA sequence reads. IGV [61] and Jalview (https://www.jalview.org/ accessed on 23 July 2020) were used to visualize the variants and haplotype-blocks detected after the analyses.

### 2.9. Annotation of Protein-Coding Sequences, Known Non-Coding RNAs and Structural RNAs

The *L. major* Friedlin genome, assembled in this work, was annotated using Companion web server (https://companion.sanger.ac.uk/ accessed on 23 April 2019) with default settings. The *L. major* Friedlin strain genome (LmjF) was used as a reference template. OrthoMCL [62] and BLAST software were used to further improve the gene annotations. Finally, the annotations were combined and used to create a GFF3 file using an in-house script in Python. 

The automatic ID codes generated by Companion were maintained. The code structure was LMJFC_XXYYYYYYYY, in which the label LMJFC is common to all annotated elements, XX stands for the chromosome number and the set of Y corresponds to a serial number, starting from 5000 at the beginning of the chromosome and increasing by 100 units for the ID of the downstream-annotated element. For structural RNAs, the nomenclature for IDs was modified to indicate the RNA type (rRNA, tRNA or snoRNA), intercalated between the chromosome number and the serial number (LMJFC_XX.rRNA.YYYY).

### 2.10. Transcriptome Definition and Annotation

Poly-A+ RNA from *L. major* promastigotes was used for library construction and Illumina sequencing (HiSeq 2000 technology); details about RNA-seq data have been described previously [63]. A total of 88,315,069 (2 × 76-nt) stranded RNA-seq reads were used for transcriptome definition. Transcripts were generated from RNA-seq reads following the pipeline developed by Rastrojo and co-workers [34]. Briefly, quality-filtered RNA-seq reads were mapped to the de novo genome assembly generated in this work (LMJFC genome) using Bowtie2 aligner (parameters: --np 0 --n-ceil L,0,0.02 --rdg 0,6 --rfg 0,6 --mp 6,2 y --score-min L,0,−0.24). Then, the assembly of transcripts was performed using Cufflinks with default parameters [64]. Additionally, among those unaligned reads, a search for the presence of eight or more nucleotides identical to the 3′-end of the SL sequence (AACTAACGCT ATATAAGTAT CAGTTTCTGT ACTTTATTG) was performed. After trimming the SL-containing reads, the remaining sequences were mapped back to the LMJFC genome to define the SL-addition sites (SASs) and, therefore, the transcript start. Using a similar strategy, i.e., searching for unaligned reads containing a poly-A stretch (>5 nt in length) at their 3′-end, the poly-A addition sites (PASs) were defined. To increase the identification of PASs, we further used the huge amount of Illumina RNA-seq reads generated by Dillon and co-workers [65] from *L. major* (Friedlin) promastigote RNA samples. In this manner, most transcripts could be precisely delimited after mapping the SASs and PASs. Finally, annotated CDSs (see above) were associated with the corresponding transcripts, which were named according to the CDS code, but intercalating a ‘T’ between the chromosome number and the CDS serial number (LMJFC_XXTYYYYYYYY). For those transcripts lacking an associated CDS, the intermediate serial number between the neighboring CDS-containing transcripts was used for naming them.

### 2.11. Generation and Annotation of Gene Models

The CDS and transcripts coordinates were merged in order to create gene-models. For simplicity, genes maintained the corresponding transcript names, but excluding the ‘T’ from the transcript ID. A final manual revision of the annotations was carried out by parallel IGV visualizations of CDSs, transcripts, and RNA-seq reads distribution on the final LMJFC genome assembly.

### 2.12. Data Availability

Genomic and transcriptomic raw reads have been deposited in the European Nucleotide Archive (ENA; http://www.ebi.ac.uk/ena/ accessed on 28 July 2020). Besides, the assembled genome and transcriptome sequences together with annotations files were uploaded under the Study accession number PRJEB25921. Additionally, the genome (fasta file), the annotations for the genome, transcriptome and gene models are downloadable at the Leish-ESP website (http://leish-esp.cbm.uam.es/ accessed on 23 August 2021).

## 3. Results and Discussion

### 3.1. Re-Sequencing and De Novo Assembly of the L. major (Friedlin Strain) Genome 

As described above, the *L. major* genome was the first to be sequenced among species of the genus *Leishmania* [7]. This fact, together with the robustness of the assembly attained, justifies that this genome became a reference in the field of trypanosomatids. Nevertheless, after its publication, a few studies documented the existence of some inaccuracies in this reference genome assembly, mostly associated with the abundance of both tandemly reiterated genes and retroposon-derived repeated sequences in the *Leishmania* genomes [19,20]. Hence, given the relevance of this genome in the field, the aim of this work was to re-assemble the genome for this strain, exploiting recent advances in sequencing technology. Recently, we have succeeded in re-assembling the genomes for two other *Leishmania* reference species, *L. infantum* [29] and *L. braziliensis* [27], and the de novo assembly of the *L. donovani* (HU3 strain) genome [30] by using the PacBio single-molecule real-time (SMRT) sequencing technology [44].

**Figure 1:**
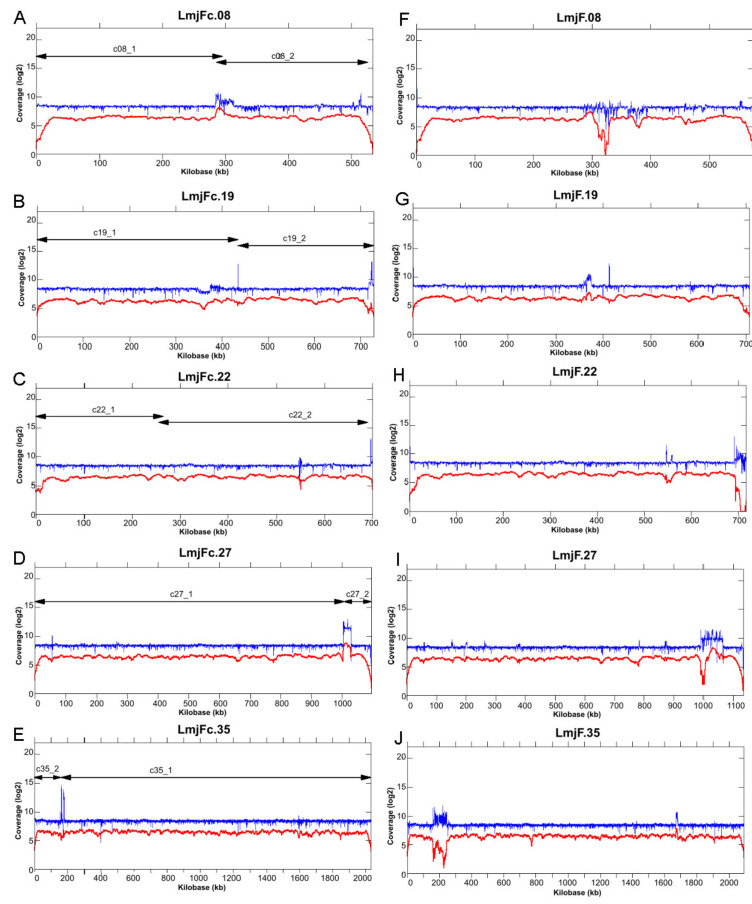
Read coverage along five chromosomes that resulted from joining two contigs. Illumina reads (in blue) and PacBio reads (in red) were mapped to the de novo assembled chromosomes (LmjFc) 8 (panel **A**), 19 (**B**), 22 (**C**), 27 (**D**) and 35 (**E**), and to the corresponding chromosomes (panels **F**–**J**) from the reference *L. major* genome (LmjF). The lines with arrow-heads denote the position of the two contigs joined to form the final chromosomes.

A total of 285,082 high-quality reads with an average length of 16-kb were generated by PacBio sequencing, representing an estimated 140-fold coverage based on the 32.8-Mb size for the *L. major* genome [7]. As detailed in Section 2, HGAP3 and HGAP4 assemblers were used to construct contigs from the PacBio reads. After filtering out contigs with low coverage and short length, a total of 41 contigs were further analyzed: 31 of them represented complete chromosomes, whereas chromosomes 8, 19, 22, 27, and 35 appeared assembled in two contigs each. However, the pairs of contigs were easily joined by Minimus2 software [47]. Figure 1 shows the reads coverage along the five chromosomes that resulted from the joining of two contigs; the coverage of both PacBio and Illumina reads was continuous along the chromosomes, indicating that the assembly of these five chromosomes was correct. Illumina sequence reads (370-fold coverage) were also generated from the same *L. major* DNA sample and used for refinements of the final nucleotide sequence and to extend chromosomal ends. In particular, 27 out of the 36 chromosomes were extended using contigs assembled from the Illumina reads; as shown in Figure 1, PacBio reads coverage drastically decreased at the chromosomal ends, suggesting structural constraints of the telomeres that affect PacBio library preparation. Additionally, the higher accuracy of Illumina reads served to correct 1964 indel errors (1894 insertions and 70 deletions) in the sequence assembled from PacBio reads by using ARAMIS [51].

Among the five chromosomes that were initially assembled in two contigs, the presence of large, repeated regions in four of them would be the cause of halting the assembly progression. No obvious structural reason could be deduced to explain that chromosome 22 were initially assembled in two contigs. In particular, the two contigs forming chromosome 27 were stopped at the rDNA locus, composed of repetition units of about 20-kb [66]. Moreover, the sudden increase of reads coverage in the rDNA locus, observed after mapping of either PacBio or Illumina reads (Figure 1D), would indicate that a sequence collapse remains yet in the final assembled chromosome 27. According to the read coverage on the assembled rDNA region regarding the median value along the entire chromosome, it was calculated that 15–16 rDNA units must exist in the locus. However, the assembled genome attained in this work (hereinafter named LMJFC) contains only two units, whereas 6 rDNA units are found in the current reference *L. major* genome assembly (LmjF). In a classical study on the *L. major* (Friedlin strain) rDNA locus, Martínez-Calvillo and co-workers estimated in 10–14 the number of rDNA units per chromosome [66]. The collapse assembly of the rDNA locus in the assembled LMJFC genome is expected, taking into account that the size of an rDNA unit is close to the mean size of the PacBio reads. Therefore, larger sequence reads would be needed to accurately determine the real number of rDNA units existing in the *Leishmania* genomes.

**Figure 2:**
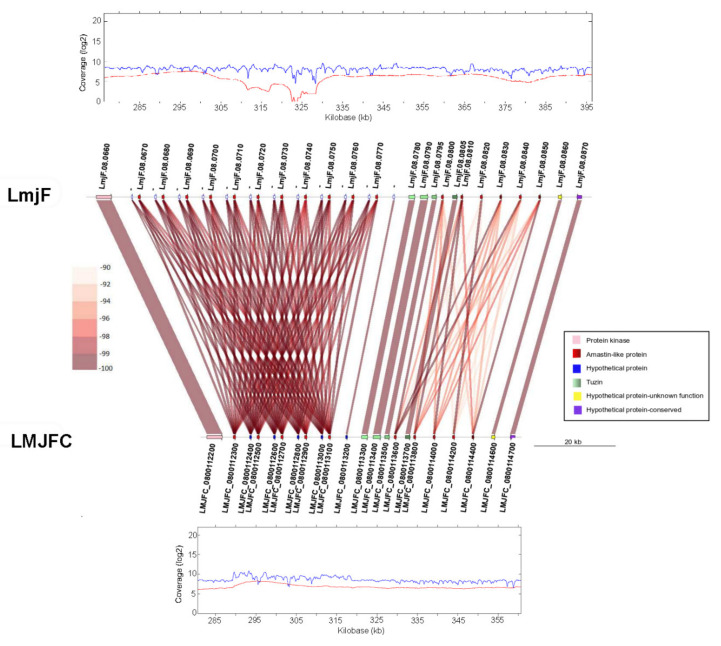
Analysis at the gene level of the differences existing between the LmjF and LMJFC assemblies in the middle of chromosome 8. LmjF corresponds to the gene organization existing in the current reference genome, whereas LMJFC corresponds to the equivalent region in the newly assembled genome. Gene sequence identity is shown according to a color-intensity scale (brow hue ranges from 90 to 100% of sequence identity). The upper and bottom graphs show the coverages of Illumina reads (in blue) and PacBio reads (in red) mapped to this chromosomal region in the LmjF and LMJFC assemblies, respectively.

Another feature in Figure 1 that caught our attention was the sudden decrease in the coverage of PacBio reads on the LmjF.08 chromosome assembly (panel F), suggesting the existence of a clear discrepancy in relation to the new assembly (LMJFC; panel A). In order to further analyze this finding, the genomic regions from both assemblies were analyzed at a per gene level (Figure 2). When PacBio sequence reads were aligned against both assemblies, a lack of coverage was observed around coordinate 323-kb of chromosome LmjF.08, indicating that this region in the LmjF genome would be misassembled. In contrast, a continuous and smooth distribution of the PacBio reads was observed when they were mapped against the LMJFC assembly (Figure 2, bottom panel). Two differences were found between both genome assemblies. Firstly, the LmjF assembly contains 11 copies of an amastin-like protein coding gene, whereas only 5 genes were assembled in the LMJFC genome. In fact, this region is composed by a repeated unit consisting in two alternating genes (amastin-like- and hypothetical protein-coding genes). The genes coding for this hypothetical protein, although existing in the LmjF.08 chromosome sequence, were not annotated previously. The other difference (minor) is that a tandem consisting of five amastin-like coding genes was assembled in the LmjF genome (IDs: LmjF.08.0810 to LmjF.08.0850) whereas only four genes comprise this tandem in the LMJFC assembly (Figure 2).

**Figure 3:**
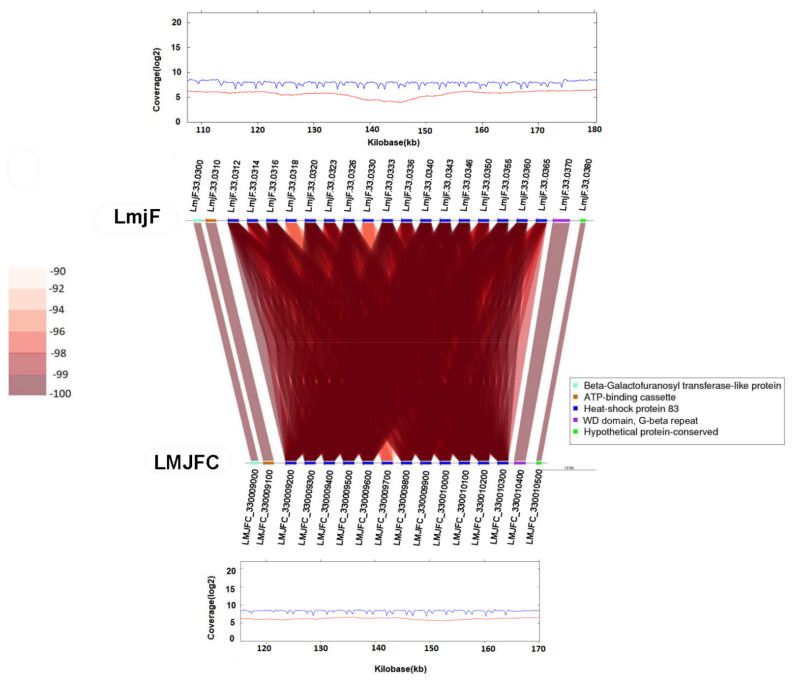
Different gene copy number in the HSP83/90 *locus* are assembled in either LmjF or LMJFC chromosome 33. Gene sequence identity is shown according to a color-intensity scale. The upper and bottom graphs show the coverages of Illumina reads (in blue) and PacBio reads (in red) mapped to this chromosomal region in the LmjF and LMJFC assemblies, respectively.

Another chromosomal region in which the LmjF assembly contains more genes than those found in the LMJFC one is the HSP83/90 locus (Figure 3). In the LmjF genome, there were annotated 17 HSP83/90 genes, but it is likely that the real number, according to the reads coverage, would be 12, as annotated in the LMJFC assembly. 

**Figure 4:**
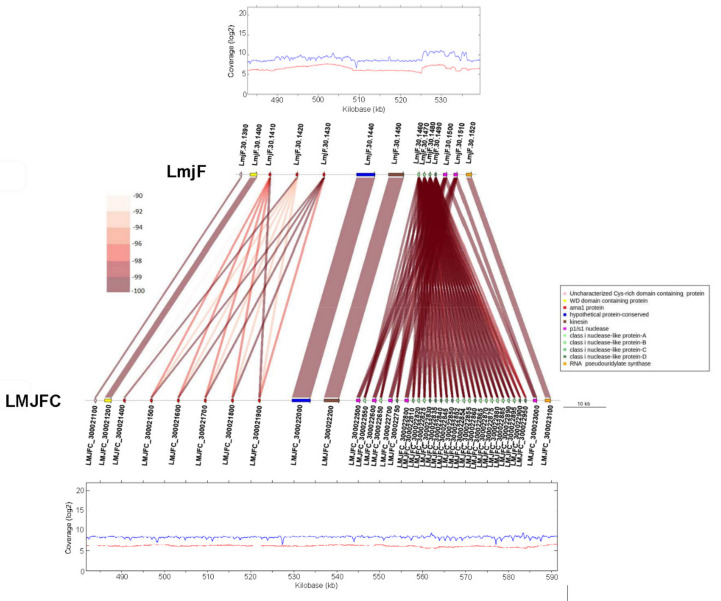
Different gene copy numbers exist in a central region of chromosome 30 in the LmjF and LMJFC assemblies. Gene sequence identity is shown according to a color-intensity scale. The upper and bottom graphs show the coverages of Illumina reads (in blue) and PacBio reads (in red) mapped to this chromosomal region in the LmjF and LMJFC assemblies, respectively.

In addition to chromosomal *loci* in which the number of repeated genes appeared as overestimated in the LmjF assembly, in some other *loci* the situation was the converse. Figure 4 illustrates a region of chromosome 30 in which both assemblies are markedly different. On the one hand, in the LMJFC assembly, six genes coding for Ama1 protein were annotated, whereas only three are found in the LmjF assembly. On the other hand, in a region located downstream of the Ama1-protein locus, the number of genes coding for a family of class i-nuclease-like proteins was found to be larger in the LMJFC assembly (24 genes) than in the LmjF genome (4 genes). Moreover, in the LMJFC assembly, there were assembled four p1/s1 nuclease-encoding genes, whereas only two are present in the LmjF genome. The fair distribution of sequencing reads on the LMJFC assembly (Figure 4, bottom), but uneven on the LmjF one (Figure 4, upper) supports that the assembly attained for *L. major* (Friedlin) chromosome 30 in this work would be closer to the real one.

**Figure 5:**
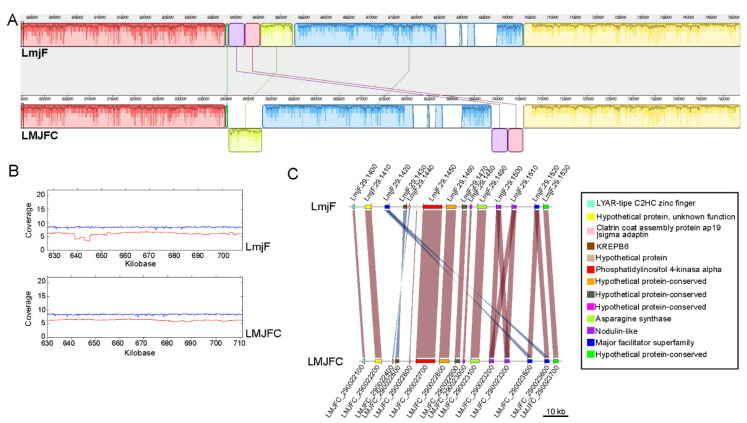
Reorganization of a region in chromosome 29 according to the LMJFC assembly regarding the reference LmjF genome. (**A**) Synteny blocks (represented by different colors that identified the conserved genomic regions) determined by pairwise comparisons between the LmjF genome (upper scheme) and the newly assembled LMJFC genome (bottom scheme), using the MAUVE tool. Blocks located underneath the *X*-axis denote inversion events. (**B**) The upper and bottom graphs show the coverages (log_2_) of Illumina reads (in blue) and PacBio reads (in red) mapped to this chromosomal region in the LmjF and LMJFC assemblies, respectively. (**C**) Schemes show the reorganization at per gene level. Genes with sequence identity and identical orientation are colored in brown, whereas blue hues were used to denote an inverted orientation between the LmjF and LMJFC assemblies.

The MAUVE tool [57] was used to visualize changes in synteny between the two *L. major* genome assemblies. Interestingly, few alterations were found, and the most remarkable one is that illustrated in Figure 5. In this region of chromosome 29, two inverted segments were found. According to the LMJFC assembly, the gene LmjF.29.1420 would be inverted and mislocated in current LmjF genome, and its real position would be adjacent to another identical gene copy (LmjF.29.1520). The IDs for these genes in the LMJFC genome are LMJFC_290023500 and LMJFC_290023600 (Figure 5, panel C). Also, genes LmjF.29.1430 and LmjF.29.1440 were found to be inverted in the LMJFC genome (IDs LMJFC_290022500 and LMJFC_290023400, respectively). The correctness of the LMJFC assembly in this region is supported by the smooth distribution of both PacBio and Illumina sequence reads, a fact contrasting with the abrupt decrease in coverage when these sequencing reads were aligned to the LmjF genome (Figure 5, panel B).

In addition, the seven genomic regions documented as absent from the reference *L. major* genome (LmjF) by Alonso et al [20] were verified in the new assembly (LMJFC); these findings reinforce the improving of the assembly attained in this work regarding the reference genome currently available.

Table 1 summarizes some features (metrics) of the *L. major* (Friedlin) genome assembled in this study (LMJFC), and how they have varied regarding the current genome available at TrytripDB (LmjF). To note, the LMJFC assembly does not contain any sequence gap and nucleotide uncertainties, which, even though in low numbers, remained in the LmjF genome. A remarkable difference between both assemblies exists in the number of annotated genes, whereas 9847 genes (excluding pseudogenes, listed in Supplementary File 1, Table S1) have been annotated in the LMJFC genome, the annotated genes in the LmjF genome (version 44) are 9293. However, most of the differences are due to the use of different annotation procedures, as many of the newly annotated genes in the LMJFC genome could be also annotated in the LmjF genome sequence. Thus, for annotations on the LMJFC genome, apart from the automatic annotation generated by Companion, a manual curation was carried out in order to incorporate genes annotated in the genomes of other *Leishmania* species and related trypanosomatids. In fact, in a strict sense, only 183 genes (mainly protein-coding genes) may be categorized as new genes, as they exist only in the LMJFC assembly (see Supplementary File 1, Table S2). Another source contributing to increase the total number of annotated genes in the LMJFC genome is the existence of two or more copies for 74 genes that were single-copy genes in the LmjF assembly (see Supplementary File 1, Table S3). On the contrary, a hundred of protein-coding genes annotated on the LmjF assembly were not maintained in the new assembly (LMJFC) due to either an excessive copy number or lack of perfect matching with sequences in the LMJFC genome (these genes are listed in Supplementary File 1, Table S4).

**Figure 6:**
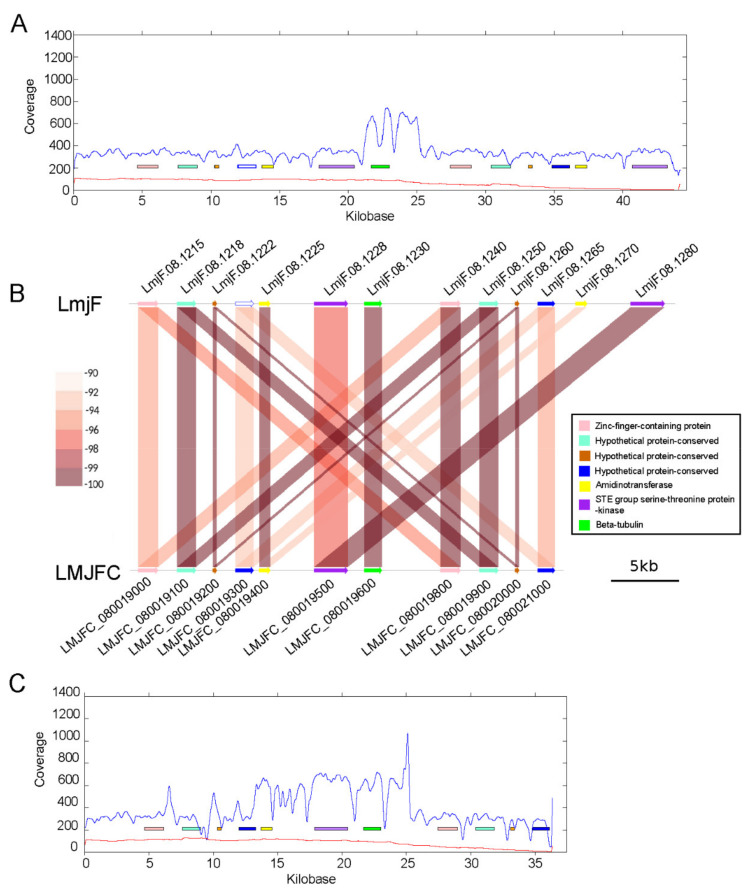
The right end of chromosome 8 has not been assembled in a definitive manner. (**A**) Linear coverages of Illumina reads (in blue) and PacBio reads (in red) mapped to this chromosomal region in the LmjF assembly. (**B**) Schemes show the gene organization and sequence identity of the genes annotated at the right end of chromosome 8 in the LmjF and LMJFC assemblies. Gene sequence identity is shown according to a color-intensity scale (brow hue ranges from 90 to 100% of sequence identity). (**C**) Linear coverages of Illumina reads (in blue) and PacBio reads (in red) mapped to this chromosomal region in the LMJFC assembly.

Although the *L. major* genome sequence has been substantially improved after the re-assembling carried out in this work, we realized that a few chromosomal regions might not be assembled in a definitive manner. Apart from the rDNA locus (discussed above, and Figure 1D), it was apparent that the 3′-end sequence of the chromosome 8 should be extended to accommodate the excess of Illumina reads mapping in this region (Figure 6, panels A-C). This region contains a block of four genes that are tandemly repeated: genes LMJFC_080019000 to LMJFC_080019300 have high sequence identity with genes LMJFC_080019800 to LMJFC_080020100 (Figure 6, panel B). According to the Illumina reads coverage, this region was found to be more accurately assembled in current LmjF genome, in which the repeated block consists of six genes (the additional genes are LmjF.08.1270 and LmjF.08.1280; Figure 6B). Even more, the Illumina reads coverage on the LmjF and LMJFC assemblies were indicating that the beta-tubulin gene also forms part of the repeated block. Remarkably, this region in the *L. donovani* (HU3 strain) chromosome 8 [30] contains two times the block with these seven genes (from the gene coding for the Zn-finger-containing protein to the gene coding for the beta-tubulin).

A question that may arise is whether some of the differences existing between the LmjF and LMJFC assemblies are due to the evolution of this strain through its axenic cultivation. Although it cannot be excluded this possibility, we think that is very improbable that the genomic reorganizations illustrated in Figure 2, Figure 3, Figure 4 and Figure 5 are the result of an evolution of the strain in our laboratory. Our thinking is based on the remarkable conservation of gene order (synteny) existing between the different species of the genus *Leishmania*. Nevertheless, the precise answer would be only obtained after sequencing in parallel different laboratory lines for this strain.

**Figure 7:**
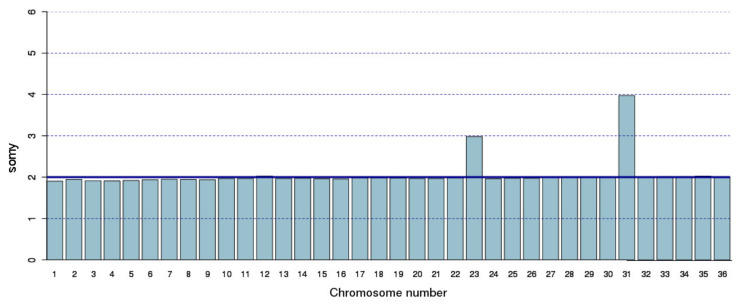
Somy analysis of the 36 chromosomes composing the *L. major* (Friedlin strain) genome. The somy values were calculated from the coverage of Illumina reads mapped to the LMJFC assembly, as detailed in Methods section. Graphs were generated with R, using the barplot function (https://cran.r-project.org accessed on 27 May 2019).

The somy of the chromosomes was calculated based on the Illumina reads coverage using the 2-loop method [56]. Most of the chromosomes in this strain were found to be diploid (Figure 7) with the exception of chromosome 23 and 31 that appeared as trisomic and tetrasomic, respectively. This karyotype is very similar to that reported for this strain by Rogers et al [18], the sole difference was that chromosome 23 was reported as diploid and, according to our calculations, the somy of this chromosome would be triploid.

**Figure 8:**
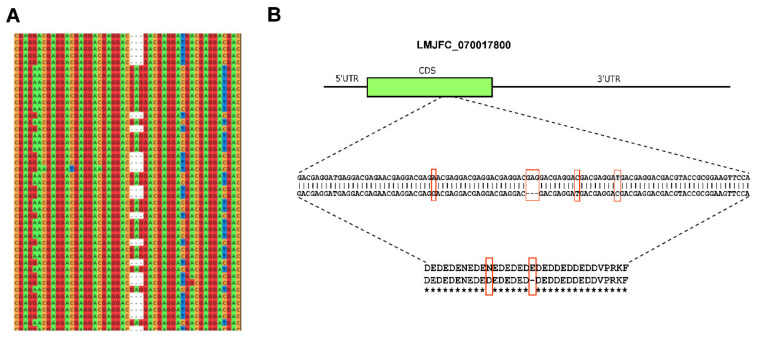
Identification of two distinct alleles for LMJFC_070017800 gene. (**A**) Two haplotype blocks were identified by the HapCUT2 tool and confirmed when Illumina reads mapping to this gene were visualized. (**B**) Scheme of gene structure, location of allelic polymorphisms and differences in the amino acid sequences between both alleles.

Additionally, we searched for allelic polymorphisms in the assembled LMJFC genome by using HapCUT2 software (see Materials and Methods for further details). A total of 2904 positions were found to be polymorphic; listed in Supplementary File 1, Table S5. Most were found to be nucleotide variations (Single nucleotide polymorphisms, SNPs), but insertions and deletions (InDels) were also frequent. Additionally, as the HapCUT software allows reconstructing individual haplotypes in diploid genomes [60], we looked for possible haplotype blocks, and 138 were indeed identified. The term haplotype block refers to a combination of consecutive variant sites (SNPs and/or small InDels) that are linked in a single chromosome. The delimitation of these haplotype blocks adds valuable information regarding the gene structure, as this allows for deducing the precise sequence of the two allelic genes co-existing in the genome. Figure 8 illustrates a haplotype block mapping on gene LMJFC_070017800. This block is constituted by three nucleotide transitions and one InDel of three nucleotides. As expected for a disomic chromosome, around 50% of the Illumina DNA-seq reads mapping on this region correspond to each allele (Figure 8, panel A). Although two of the SNPs represent silent changes, the other SNP and the InDel would be suggesting a possible co-existence of two proteins, differing each other in two amino acids (Figure 8, panel B). If this change in the sequence has a functional role that merits being analyzed, in view of a recent article in which a single amino-acid substitution in the *L. donovani* RagC protein was found to dramatically affect the virulence of this parasite [67]. The gene LMJFC_070017800 was annotated as coding for a putative nucleolar RNA-binding protein, but no additional studies on this protein have been reported to date.

### 3.2. Transcriptome of L. major Friedlin Strain Based on the New Assembly (LMJFC)

The first annotated transcriptome for a species of the genus *Leishmania* was generated by our group, and that was the transcriptome for the *L. major* Friedlin strain based on the reference LmjF genome [34]. Here, we have refined the transcriptome of this strain, using the new genome assembly (LMJFC) and RNA-seq data derived from both previous [34] and more recent [63] studies. Table 2 summarizes the main features of the *L. major* transcriptome. A total of 9828 transcripts were annotated in the LMJFC genome, and the complete list is provided in the Supplementary File (Table S6). The vast majority of the annotated coding sequences (CDS) are associated with defined transcripts, but transcripts could not be delimited for the following annotated CDS: LMJFC_020007050, LMJFC_020009550, LMJFC_070019450, LMJFC_090013750, LMJFC_100007750, LMJFC_170022050, LMJFC_270007950, LMJFC_270026300, LMJFC_280034650, LMJFC_290011550, LMJFC_310033150, LMJFC_350010450 and LMJFC_350034300. In these cases, Cufflinks software failed in generating transcripts due to the very low number of RNA-seq reads mapping to these genes, suggesting that they are not expressed in the *L. major* promastigote form. On the other hand, 47 transcripts were categorized as polycistronic (43 of them bicistronic), as they contained two or more annotated CDS. Further experimental approaches will be required to determine whether these CDS also exist as individual transcripts.

A common feature to most of the *Leishmania* transcripts is the presence of the 39-nucleotides SL sequence at their 5′-end. In our study, this sequence was found in the vast majority of the transcripts (Table 2). Moreover, for 9341 out of the 9828 annotated transcripts, a multiplicity of SL addition sites was evidenced. This finding means that around 95% of the genes are transcribed into two or more RNA species differing in the length of their 5′-UTRs. In the Supplementary File (Table S6) a complete list of main and alternative SL addition sites (SASs) for every gene is provided. At the 3′-end, transcripts were delimited based on the presence of a non-encoded poly-A tail. Thus, the poly-A addition site (PAS) was identified in 8677 of the annotated transcripts. Again, a heterogeneity in the PAS usage was evidenced, as 6668 of those transcripts were found to be polyadenylated using two or more alternative PAS.

**Figure 9:**
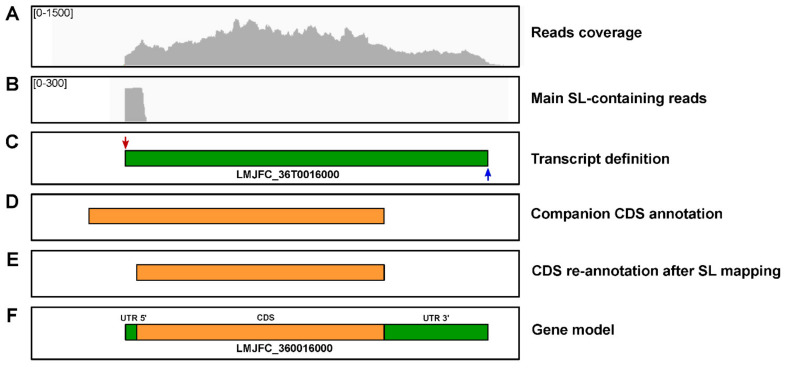
Process followed in the generation of the gene models. (**A**) RNA-seq reads distribution. (**B**) Mapping of RNA-seq containing SL-sequences. (**C**) Definition of the transcript boundaries based on the SAS (supported by 213 reads) and PAS (4 reads) positions. (**D**) CDS as annotated by Companion. (**E**) CDS re-annotated according to SAS position. (**F**) Final gene model for LMJFC_360016000 gene.

Additionally, the analysis of SAS allowed correction of misannotated CDS. As mentioned above, CDS annotation was done automatically on the LMJFC genome sequence by the Companion tool [32]. This software is designed to annotate a new genome based on a reference genome (in our case, we selected the LmjF genome). When the SASs detected in this study were positioned relative to the automatically annotated CDSs, we found that main SASs were located, in some cases, within the predicted CDS. Therefore, CDS annotation of those genes had to be shortened in order to establish the first in-phase ATG, downstream from the SAS, as the initiation codon. This modification was introduced for 247 genes. An example, based on gene LMJFC_360016000, is shown in Figure 9. This figure also illustrates the process followed in the definition of the gene models that consisted of four steps: (i) the transcripts are created from the distribution of RNA-seq reads; (ii) transcripts are trimmed at their ends by mapping SAS and PAS; (iii) Companion-annotated CDS are placed on the transcript; (iv) a gene model including 5′- and 3′-UTR is created. All the gene models generated in this study are listed in the Supplementary File (Table S7). 

The availability of gene models is crucial for analyzing gene expression, either in studies dealing with individual genes or in projects following whole-genome strategies. Thus, the ectopic expression of a given gene may vary according to the regulatory sequences surrounding the coding sequence and this may explain that phenotypic defects in the deletion mutant could be not restored by add-back plasmids containing the coding regions without their regulatory sequences [42]. On the other hand, *Leishmania* genomes contain many repeated genes having identical CDS but different UTRs. In this situation, when expression studies are conducted at the genomic-scale based solely in CDS coordinates, it is not possible distinguish whether differential expression exists among the repeated genes [63].

Upon establishment of gene models, we used RNA-seq data from a recent study [63] to quantify relative transcript levels in *L. major* promastigotes. The measuring of the relative RNA abundance was carried out by the TPM (transcripts per million) method [68]. A wide range of expression levels was observed, from 3660 TPM for transcript LMJFC_27T0019600 to near zero (transcript LMJFC_36T0057500). In Table S8 (Supplementary File), the relative levels for every one of the transcripts delineated in this study are listed. Table 3 shows the 40 transcripts with the highest expression levels. Two transcripts coding for histone H1 were found to be the most expressed, and 11 additional transcripts coding for nucleosomal histones (H2A, H2B, H3, and H4) were ranked among the top 40 expressed genes. This is an expected finding given the abundance of these proteins in the cell. In addition, half of the more expressed genes code for ribosomal proteins, again abundant cellular constituents. Therefore, it is worthy to discuss about the presence of the other transcripts listed among the most abundant poly-A+ RNA molecules in the *L. major* promastigotes. The sixth most abundant transcript (LMJFC_36T0028500) codes for an inosine-guanosine transporter named NT2. Purine transporters are essential in *Leishmania* and other trypanosomatids, since they are incapable of purine biosynthesis and must acquire purines from the host milieu [69]. The ninth transcript in the list is LMJFC_13T0009700, which codes for ALBA protein 1. Alba proteins are RNA-binding proteins that participate in mechanisms controlling developmentally regulated gene expression and have been reported to regulate translational efficiency and turnover rate of particular transcripts in *Leishmania* [70,71]. A transcript coding for a nucleoside diphosphate kinase (LMJFC_32T0040100) occupied the twenty-first position, which also agrees with the relevant role played by these enzymes, i.e., they catalyze the transfer of the γ-phosphate moiety from a nucleoside triphosphate (NTP) donor to an NDP acceptor in order to maintain appropriate cellular levels of NTPs [72]. The 25th transcript (LMJFC_25T0016400) encodes for a cyclophilin having peptidyl-prolyl cis/trans isomerase activity [73]; this isomerase activity is essential for protein folding after translation. Another abundant transcript, LMJFC_32T0014200 (position 29th; Table 3) encodes for a protein-binding protein; the homologous protein in the *Leishmania*-related trypanosomatid *Trypanosoma cruzi* has been described as an abundant protein associated with polysomes [74]. Finally, position 39^th^ is occupied by transcript LMJFC_35T0029900, which codes for the kinetoplastid membrane protein 11 (KMP11); accordingly, several millions of KMP11 molecules have been estimated to exist per promastigote cell [75]. In summary, according to the functional relevance and literature data, all the transcripts listed in Table 3 would be coding for abundant *Leishmania* proteins. In this regard, in the list, there is a transcript (LMJFC_31T0016500, ranked 35^th^) that encodes for a short protein (79 amino acids in length) of unknown function. This protein is well-conserved among different trypanosomatids, and according to the expression levels of its transcript, might be also a highly abundant molecule in *L. major* promastigotes.

## 4. Conclusions

The combination of the second (Illumina Inc., San Diego, CA, USA) and third (PacBio, Silicon Valley, CA, USA) NGS tools proved to be a powerful strategy for attaining complete assemblies of genomes. In this work, based on the use of both technologies, a de novo assembly of the *L. major* reference strain (Friedlin) genome is reported. Although the previous reference genome for this strain (LmjF; [7]) may be categorized as an outstanding assembly and has been widely used for a long time, the assembly attained in this work represents an improved version that should replace the LmjF genome. It should be borne in mind that genomic-whole studies, either transcriptomics or proteomics, depend on the accuracy of the sequence and annotations of the reference genome.

Another contribution of this work is the generation of the poly-A+ transcriptome for the *L. major* promastigote stage. Essentially, all the protein-coding genes are represented in the transcriptome. Moreover, remarkable heterogeneities in the SL and polyadenylation sites were observed for around 95% of the transcripts. The combination of gene annotations and transcript delimitation have allowed the generation of gene models for the entire *L. major* genome. A precise determination of the 5′- and 3′-UTRs is mandatory for studies dealing with gene expression, mainly in organisms like *Leishmania*, in which gene expression regulation occurs almost exclusively at the post-transcriptional level.

To our knowledge, this is the first report in which the existence of haplotype blocks has been analyzed in the *L. major* genome. The coexistence of different alleles for a given gene adds another layer of complexity that might have phenotypic implications in this parasite. 

## Figures and Tables

**Table 1:** Parameters in the new (LMJFC) and previous (LmjF) genome assemblies.

Parameters/Genome	LMJFC [This Work]	LmjF [v44-TritrypDB]
Number of chromosomes	36	36
Protein-coding genes	8596	8400
Pseudogenes	88	88
rRNAs	30	63
tRNAs	93	83
snoRNA+snRNA+slRNA	1128	747
Number of gaps	0	9
Number of Ns	0	13
Genome size (bp)	32,792,963	32,855,082

**Table 2:** The poly-A+ transcriptome of *L. major* Friedlin strain.

Annotated Transcripts	9828
Protein-coding transcripts	8517
Transcripts with mapped SL addition (SLA) site	9745 (99.1 %)
Transcripts with alternative SLA sites	9341 (95 %)
Transcripts with mapped poly-A addition site (PAS)	8677/9336 ^1^
Transcripts with alternative PASs	6668
Annotated CDS lacking a defined transcript	10
Transcripts with two or more CDS	47 (43 are bicistronic)

^1^ PAS mapped after analysis of RNA-seq raw data, publically available, produced by Dillon et al [65].

**Table 3:** The 40 most abundant transcripts in L. major (Friedlin strain) promastigotes.

Transcript ID	TPM ± SD ^1^	Name of the Encoded Protein
LMJFC_27T0019600	3660.01 ± 344.91	histone H1
LMJFC_27T0019100	3629.05 ± 386.87	histone H1
LMJFC_35T0007800	3477.13 ± 722.67	ribosomal protein L30
LMJFC_06T0005100	3034.31 ± 646.98	histone H4
LMJFC_29T0026200	3027.80 ± 656.33	histone H2A
LMJFC_36T0028500	2876.01 ± 261.79	inosine-guanosine transporter (NT2)
LMJFC_19T0005400	2862.36 ± 308.82	histone H2B
LMJFC_19T0005500	2684.51 ± 221.06	histone H2B
LMJFC_13T0009700	2578.58 ± 230.03	ALBA-domain protein 1 (ALBA1)
LMJFC_19T0005600	2555.44 ± 211.16	histone H2B
LMJFC_30T0045400	2430.21 ± 261.54	ribosomal protein L9
LMJFC_15T0005100	2294.60 ± 265.01	histone H4
LMJFC_16T0012400	2285.04 ± 250.45	histone H3
LMJFC_35T0048600	2246.30 ± 380.22	ribosomal protein L23
LMJFC_31T0048800	2078.23 ± 130.10	histone H4
LMJFC_29T0026000	2068.24 ± 167.92	histone H2A
LMJFC_24T0032000	2060.45 ± 60.25	ribosomal protein L12
LMJFC_19T0005700	2057.42 ± 51.88	ribosomal protein S2
LMJFC_13T0011100	2052.88 ± 103.60	ribosomal protein S12
LMJFC_09T0020600	1994.99 ± 193.32	histone H2B
LMJFC_32T0040100	1964.77 ± 53.15	nucleoside diphosphate kinase b
LMJFC_35T0011400	1939.67 ± 357.53	ribosomal protein L18a
LMJFC_28T0032200	1935.03 ± 70.94	ribosomal protein S29
LMJFC_35T0026300	1899.65 ± 350.17	ribosomal protein L15
LMJFC_25T0016400	1898.52 ± 326.43	cyclophilin A | CyP1
LMJFC_29T0039100	1898.13 ± 80.33	ribosomal protein S19-like protein
LMJFC_14T0020500	1887.76 ± 63.85	ubiquitin/ribosomal protein S27a
LMJFC_32T0010100	1857.84 ± 87.17	ribosomal protein L17
LMJFC_32T0014200	1836.37 ± 60.13	RNA binding protein
LMJFC_32T0010300	1833.43 ± 53.95	ribosomal protein S2
LMJFC_35T0027400	1814.80 ± 300.02	ribosomal protein S6
LMJFC_36T0051800	1798.18 ± 96.35	ribosomal protein L34
LMJFC_35T0042900	1794.95 ± 276.06	ribosomal subunit protein L31
LMJFC_32T0016000	1782.48 ± 98.87	ribosomal protein L18a
LMJFC_31T0016500	1780.58 ± 476.99	hypothetical protein-conserved
LMJFC_35T0048200	1779.35 ± 288.58	ribosomal protein L27A/L29
LMJFC_29T0034600	1779.35 ± 55.64	ribosomal protein L13
LMJFC_35T0048400	1770.18 ± 217.83	ribosomal protein L27A/L29
LMJFC_35T0029900	1754.88 ± 188.03	kinetoplastid membrane protein 11 (KMP11)
LMJFC_25T0035900	1745.73 ± 104.64	histone H4

^1^ Standard deviation (SD).

## Data Availability

Genomic and transcriptomic raw reads have been deposited in the European Nucleotide Archive (ENA; http://www.ebi.ac.uk/ena/ accessed on 20 August 2021). Besides, the assembled genome and transcriptome sequences together with annotations files were uploaded under the Study accession number PRJEB25921. Additionally, the genome (fasta file), the annotations for the genome, transcriptome and gene models are downloadable at the Leish-ESP website (http://leish-esp.cbm.uam.es/ accessed on 23 August 2021).

## Supplementary Materials

The following are available online at www.mdpi.com/article/10.3390/genes12091359/s1, Supplementary File containing: Table S1. List of putative pseudogenes found in the LMJFC genome; Table S2. New genes annotated in the LMJFC assembly that could not be found in the LmjF genome sequence; Table S3. Single-copy genes in the LmjF assembly that were found to be repeated in the LMJFC assembly; Table S4. List of genes in the LmjF assembly that are not present in the LMJFC assembly; Table S5. Polymorphic positions found in the LMJFC genome; Table S6. Transcriptome: names, coordinates, SL addition sites (SAS, both main and alternatives), poly-A addition site (PAS, both main and alternatives) and associated function of the encoded protein; Table S7. Gene models defined in the LMJFC genome; Table S8. Relative expression levels (TPM) of the transcripts annotated in the LMJFC genome.
